# Hepatic Macrophage as a Key Player in Fatty Liver Disease

**DOI:** 10.3389/fimmu.2021.708978

**Published:** 2021-12-09

**Authors:** Liyun Xu, Wen Liu, Fuxiang Bai, Yong Xu, Xiaohong Liang, Chunhong Ma, Lifen Gao

**Affiliations:** ^1^Key Laboratory for Experimental Teratology of Ministry of Education, Shandong Key Laboratory of Infection and Immunity and Department of Immunology, School of Basic Medical Sciences, Cheeloo College of Medicine, Shandong University, Jinan, China; ^2^Cell and Molecular Biology Laboratory, Zhoushan Hospital, Zhoushan, China; ^3^Institute of Basic Medicine Sciences, Shandong First Medical University and Shandong Academy of Medical Sciences, Jinan, China; ^4^Laboratory for Tissue Engineering and Regeneration, School of Stomatology, Shandong University, Jinan, China; ^5^Department of Laboratory, Yueyang Hospital, Hunan Normal University, Yueyang, China

**Keywords:** macrophages, NAFLD, TLRs, NLRP3 inflammasome, lipid toxicity

## Abstract

Fatty liver disease, characterized by excessive inflammation and lipid deposition, is becoming one of the most prevalent liver metabolic diseases worldwide owing to the increasing global incidence of obesity. However, the underlying mechanisms of fatty liver disease are poorly understood. Accumulating evidence suggests that hepatic macrophages, specifically Kupffer cells (KCs), act as key players in the progression of fatty liver disease. Thus, it is essential to examine the current evidence of the roles of hepatic macrophages (both KCs and monocyte-derived macrophages). In this review, we primarily address the heterogeneities and multiple patterns of hepatic macrophages participating in the pathogenesis of fatty liver disease, including Toll-like receptors (TLRs), NLRP3 inflammasome, lipotoxicity, glucotoxicity, metabolic reprogramming, interaction with surrounding cells in the liver, and iron poisoning. A better understanding of the diverse roles of hepatic macrophages in the development of fatty liver disease may provide a more specific and promising macrophage-targeting therapeutic strategy for inflammatory liver diseases.

## Introduction

Fatty liver disease was proposed by Schaffner et al. in 1986 (1), characterized by the over-deposition of cytoplasmic triglycerides (TGs), as lipid droplets, in the liver. Initially, fatty liver disease is classified into two categories: alcoholic fatty liver disease and non-alcoholic fatty liver disease (NAFLD). With the improvement of living standards and changes in dietary habits in recent years, risk factors, including but not limited to overweight, type 2 diabetes, and a sedentary lifestyle, have facilitated the occurrence of NAFLD as one of the most prevalent chronic liver diseases in the world, affecting 20–30% of the general population (2). Recently, NAFLD has been renamed as a consensus and appropriate nomenclature for metabolically associated fatty liver disease (MAFLD) (3), characterized by cytoplasmic TG vacuole deposition exceeding 5% of hepatocytes in the absence of other recognized sources of fatty liver (eg., drugs and viral infection) (4). MAFLD represents a broad term encompassing a spectrum of pathological conditions. The first stage is steatosis, as the most common type, and may progress from steatohepatitis to advanced liver fibrosis, cirrhosis, and ultimately hepatocellular carcinoma. Without external intervention, fatty liver disease is becoming the leading determinant of liver transplantation, liver-related morbidity, and mortality (5). Recently, the “multiple hits” hypothesis, which includes hits coming from the liver, adipose tissue adipocytokines, and gut microbiota, provides a more accurate explanation of the pathogenesis of fatty liver disease (6). In this process, hepatic macrophage-derived inflammatory mediators play key roles in the pathogenesis of fatty liver disease. However, the biochemical events involved in fatty liver disease have not been well explored due to an incomplete understanding of the complicated pathogenesis. Thus, it is important to elucidate and explore the precise pathogenesis of fatty liver disease, which may provide macrophage-targeting immune therapeutic strategies for the intervention of fatty liver disease.

The liver is not only a central organ of energy metabolism, but it also acts as an immune organ, which is rich in multiple immune cells; thus, macrophages are abundantly found in the liver. Innate immunocytes and related effector factors play an indispensable role in the development of fatty liver disease. Immunoregulation in the liver mainly occurs at the hepatic sinusoid capillary junction of the hepatic portal vein and hepatic artery branches (7), where Kupffer cells (KCs) are in close contact with hormones, bacterial endotoxins, metabolites, and immune complexes carried from the hepatic circulation. Macrophages form a highly active, dynamic, and complex immune network system that plays various vital roles in fatty liver disease progression. Three major distinct origins of macrophage subpopulations exist in the liver: yolk sac-derived tissue-resident macrophages–KCs, monocyte-derived macrophages (MDMs)/myeloid-derived mononuclear macrophages, and liver capsular macrophages (LCMs). Although the sources of hepatic macrophages show obvious heterogeneity in the liver, it is difficult to distinguish these populations based on the existing techniques. Moreover, a consensus has not been reached on classification markers, and MDMs sometimes are known to switch to KCs under pathological conditions; therefore, in this review, we have not distinguished them strictly, and most hepatic macrophages are referred to as KCs.

Over the past few years, several reviews (Baffy G. J Hepatol. 2009; Lanthier N. World J Hepatol. 2015; Lefere S et al. JHEP Reports 2019; Chen J et al. Int J Biol Sci. 2020) have summarized the key roles of macrophages in fatty liver disease in different ways, including the heterogeneity, recruitment of macrophages, and crosstalk between macrophages and metabolic stimuli, etc. (8–11). Since hepatic macrophages contribute to both inflammation and tissue homeostasis, this review provides a comprehensive update of hepatic macrophages in fatty liver disease, mainly focusing on the origins, heterogeneities, and pathways of hepatic macrophages in the pathogenesis of fatty liver disease. We expect that this review will broaden the understanding of the association between hepatic macrophages and fatty liver disease, which would shed new light on the potential application of macrophages in the intervention of fatty liver disease.

## The Origins and Phenotype Switch of Liver Macrophages

Hepatic macrophages, consisting of resident KCs, MDMs, and LCMs, display a remarkable heterogeneity (**Table 1**). The origins of liver macrophages differ greatly, and MDMs can switch to KCs. KC was named by the German anatomist Karl Wilhelm von Kupffer. In 1997, Naito et al. found that KCs originated almost exclusively from yolk sac-derived erythromyeloid progenitors in the liver of mice during the first 9.5–12.5 days after the start of embryogenesis (12, 13). These findings were confirmed by using the cell tracer technique (14). As a subpopulation of liver-resident macrophages, KCs possess self-renewal properties depending on M-CSF signaling and exert strong phagocytosis and efferocytosis (15). The recruited monocytes rapidly differentiate into pro-inflammatory M1-like macrophages in high-fat diet (HFD)-fed mice (16).

**Table 1:** Characteristics of the liver macrophage subsets.

Subsets	Origins	Phenotypes	Properties	Receptors	Functions
KCs	Yolk sac-derived erythromyeloid progenitors	CD11b^lo^F4/80^hi^ (mice)	Self-renewal	CLEC4F	Strong phagocytosis and efferocytosis
		CD163L (human)		VSIG4	
				TIM-4	
MDMs	Circulating monocytes	CD11b^+^F4/80^+^ (mice)	Differentiate into KCs	High lysozyme 2	Secrete pro-inflammatory cytokines and fibrogenic cytokines
		CLEC5A (human)			
LCMs	Blood monocytes	F4/80^+^MHCII^hi^CX3CR1^hi^			Detects peritoneal bacteria and promotes neutrophil recruitment

KCs, Kupffer cell; MDMs, mononuclear-derived macrophages; LCMs, liver capsular macrophages; CLEC4F, C-type lectin domain family 4 member F; VSIG4, V-set and Ig domain-containing 4; TIM-4, T cell immunoglobulin- and mucin-domain-containing molecule.

Infiltrating MDMs are derived from circulating monocytes. Two major populations of circulating monocytes exist in mice: lymphocyte antigen 6C^+^ (Ly6C^+^) high (Ly6C^hi^)-expressing monocytes are present in the bone marrow, and Ly6C low (Ly6C^lo^)-expressing monocytes are derived from the spleen (17, 18). Recent evidence shows that CD11b^+^F4/80^+^ macrophages originate from infiltrating monocytes, while CD11b^lo^F4/80^hi^ macrophages are derived from resident KCs and mature monocytes (19, 20).

The monocytes in mice are marked with Ly6C^hi^ and CC-chemokine receptor 2^hi^ (CCR2^hi^) (21). Several studies have suggested that infiltrated Ly6C^hi^ monocytes in early murine steatohepatitis are mainly identified by chemokine receptors, pattern recognition receptors (PRRs), and cytokine secretion (22). Ly6C^lo^ monocytes are characterized by their scavenger receptors (23). Recently, single-cell RNA sequencing has suggested that KCs are characterized by increased C-type lectin domain family 4 member F, V-set and Ig domain-containing 4, and T-cell immunoglobulin- and mucin-domain-containing molecule, whereas MDMs are mainly identified by high lysozyme 2 in the liver of murine steatohepatitis (24).

Another liver-resident macrophage subset is the LCM; distinct from KCs ontogenetically and phenotypically, LCMs are replenished from blood monocytes and are identified as F4/80^+^MHCII^hi^CX3CR1^hi^, which detects peritoneal bacteria and promotes neutrophil recruitment to the capsule (25). However, there is no consensus on the specific marker of hepatic macrophages; thus, further investigation is required to clarify the sources of macrophages.

Although KCs and MDMs show controversial markers, it should be noted that KCs and MDMs in the liver are not immutable. Under severe hepatic damage, MDMs can differentiate into KCs when KCs are depleted (26). In the livers of Western diet-induced MAFLD mice, the recruited monocytes could also be differentiated into a distinct population of KCs termed hepatic lipid-associated macrophages, characterized by osteopontin expression and a similar capacity of lipid metabolism to that in adipose tissue (27). At the early stage of liver injury, CC-chemokine ligand 2 (CCL2), which is secreted by KCs, triggers circulating Ly6C^hi^ monocytes with CCR2 recruitment into the liver, and the recruited MDMs further secrete pro-inflammatory cytokines and fibrogenic cytokines, accelerating the progression of fibrosis (28). In the later phase of liver injury, the improved phagocytic activity of macrophages facilitates Ly6C^hi^ macrophage differentiation into the Ly6C^lo^ macrophage subset and induces extracellular matrix degradation by matrix metalloproteinases (MMPs) (29). However, another study demonstrated contrasting results that Ly6C^lo^ macrophages are derived from the spleen and are not switched from Ly6C^hi^ (23). In addition, peritoneal macrophage infiltration is also manifested in liver injury (30). However, the mechanisms of peritoneal macrophage recruitment remain poorly understood.

Monocytes in humans can be identified by CD14 and CD162 (31). In humans, the surface markers of MDMs and KCs are CLEC5A and CD163L, respectively (32). In patients with fatty liver disease, a marked increase in the number of hepatic macrophages occurs gradually with the aggravation of steatosis and inflammation (15), which is mainly attributed to the extensive infiltration of CD11b^+^Ly6C^+^ monocytes into the liver.

## Polarization of Liver Macrophages

Macrophages are the most plastic cells in the hematopoietic system and show great functional diversity. Liver macrophages can switch their phenotype towards pro-inflammatory (classically activated macrophages, designated M1-like macrophages) or anti-inflammatory (alternatively activated macrophages, designated M2-like macrophages) in response to various signals, such as cytokines, fatty acids, endotoxins, metabolites, and danger-/pathogen-associated molecular patterns (DAMPs/PAMPs) (**Table 2**). Thus, liver macrophages may display a variety of or even completely opposite roles in different diseases and even in different stages of the same disease.

**Table 2:** Comparison of M1- and M2-like macrophages.

Subsets	Stimulators	Phenotypes	Secretors	Functions
M1	LPS, IL-12, IFN-γ, TNF-α, or GM-CSF, PA	IL-12^hi^IL-23^hi^IL-10^lo^, iNOS	IL-1β, IL-12, TNF-α, IL-6, CCL2, and CCL5, NO, ROS	Pro-inflammatory, anti-tumor, and anti-bacterial
M2	IL-4, IL-13, IL-33, IL-14, OA, probiotic	IL-12^lo^IL-23^lo^IL-10^hi^, Arg-1, type 2 mannose receptor	IL-10, IL-4, IL-13, and TGF-β	Anti-inflammatory response, tissue repair

LPS, lipopolysaccharide; IL, interleukin; IFN, interferon; TNF, tumor necrosis factor; GM-CSF, granulocyte–macrophage colony-stimulating factor; PA, palmitic acid; OA, oleic acid; iNOs, inducible nitric oxide synthase; ROS, reactive oxygen species; TGF, transforming growth factor.

*In vitro*, hepatic macrophages are skewed towards M1, similar to that of macrophages exposed to lipopolysaccharide (LPS), IL-12, IFN-γ, TNF-α, or GM-CSF. Activated M1-like macrophages produce a set of pro-inflammatory mediators (*e*.*g*., IL-1β, IL-12, TNF-α, CCL2, and CCL5) and increased reactive oxygen species (ROS) and nitric oxide (NO) intermediates, displaying an IL-12^hi^IL-23^hi^IL-10^lo^ phenotype, and exert pro-inflammatory, anti-tumor, and anti-bacterial effects. In contrast, M2-like macrophages, which are primed by IL-4, IL-13, IL-33, or IL-14, release IL-10, IL-4, IL-13, and TGF-β cytokines, displaying an IL-12^lo^IL-23^lo^IL-10^hi^ phenotype, triggering an anti-inflammatory response and tissue repair.

In the microenvironment of fatty liver disease, cytokines and various kinds of fatty acids regulate macrophage differentiation. The saturated fatty acid palmitic acid (PA) induces pro-inflammatory M1-like macrophage polarization through hypoxia-inducible factor 1α, identified by increased TNF-α and IL-6 production, whereas the unsaturated fatty acid oleic acid (OA) promotes anti-inflammatory M2-like macrophage differentiation, characterized by the increased expression of arginase-1, type 2 mannose receptor, and IL-10 (33). Furthermore, probiotic (eg., *Lactobacillus paracasei*) administration also increases the number of M2-like macrophages in the liver of murine steatohepatitis and alleviates steatosis (34). In addition, macrophage polarization differs between mouse strains. In C57BL/6 mice with fatty liver disease, steatosis promotes the secretion of IL-1β, which is beneficial for M1-like macrophage polarization, whereas in BALB/c mice, steatosis mainly induces M2-like macrophage responses (35).

*In vivo*, hepatic macrophages are stimulated by endotoxins, cytokines, lipids, and other metabolites; thus, phenotypes may change dynamically with the progress and development of fatty liver disease (36). In methionine- and choline-deficient (MCD) diet-induced murine steatohepatitis, a phenotypic switch is observed from M1- to M2-like macrophages, accompanied by a shift in cytokine levels (37). Some studies have also shown that hepatic macrophages seem to express biomarkers of both M1- and M2-like macrophages simultaneously in the process of liver injury (38). The evidence mentioned above suggests that macrophage polarization is a highly plastic physiological process in the progression of fatty liver disease.

Several studies have revealed that M1-like macrophages promote hepatocyte steatosis and insulin resistance (IR), whereas M2-like macrophages show the opposite effect (39). Compared with BALB/c mice, C57BL/6 mice fed an MCD diet display more severe lipid deposition and inflammation in the liver, while M2-like macrophage polarization induced by pharmaceuticals partially inhibits lipid deposition and apoptosis in hepatocytes (40). Moreover, *Arg-2^-/-^* mice develop steatosis spontaneously and exhibit the characteristics of steatohepatitis without HFD induction (41). In murine fatty liver disease, M1-like macrophages promote TG synthesis by increasing the activity of diacylglycerol (DAG) transferase (19), promoting liver inflammation through vascular cell adhesion molecule-1, intercellular adhesion molecule-1 (ICAM-1), and TNF-α (42) and by inhibiting fatty acid oxidation by peroxisome proliferator-activated receptor α (PPARα) (43). Notably, IL-10 secreted by M2-like macrophages leads to the apoptosis of M1-like macrophages and senescence of hepatocytes (40). A study of HFD-induced fatty liver disease in mice showed that macrophages with cytokine deficiency (IL-4, IL-10, and IFN-γ) are prone to polarization to the M2-like phenotype, which aggravates liver inflammation and fibrosis (44). However, it has also been reported that, in patients with steatohepatitis, differentiated M2-like macrophages increase the risk of liver fibrosis but do not promote liver tissue repair (23).

These data together highlight that the regulatory roles of M1- and M2-like macrophages are not uniform in fatty liver disease.

## Roles of Hepatic Macrophages in Fatty Liver Disease

Normally, KCs contribute to maintaining tissue homeostasis by expressing low levels of major histocompatibility complex II molecules and co-stimulatory molecules (45), high levels of programmed cell death ligand 1, and inhibitory cytokines IL-10 and TGF-β. KCs promote regulatory T cells to facilitate immune tolerance in the liver (46, 47). In contrast, KCs also recognize extrinsic antigens to induce immune responses through PRRs and complements (48). KC depletion by treatment with clodronate-encapsulated liposomes or gadolinium chloride rapidly alleviated steatosis and inflammation in fatty liver disease, probably due to the decreased expression of inflammatory cytokines and fibrosis-related genes, and diminished insulin resistance in hepatocytes (43). However, Clementi AH et al. found that, in a diet-induced obese mice model, KC ablation increased hepatic steatosis, STAT3 signaling, and additional hepatic TG accumulation (49).

Hepatic macrophages play various roles in the different stages of fatty liver disease. In the early stage of hepatic injury, Ly6C^hi^ inflammatory monocytes and neutrophils are recruited to the liver by KCs and differentiate into CD11b^+^F4/80^+^ M1-like macrophages. During acute inflammation, KCs can degrade the extracellular matrix and repair tissue injuries. During the repair period, macrophages selectively differentiate into the M2-like phenotype, which promotes fibrosis progression by secreting IL-13 and TGF-β. In summary, these findings highlight that KCs play a complex role and show functional plasticity in the progression of fatty liver disease.

## Patterns of Hepatic Macrophages Participating in the Pathogenesis of Fatty Liver Disease

**Figure 1:**
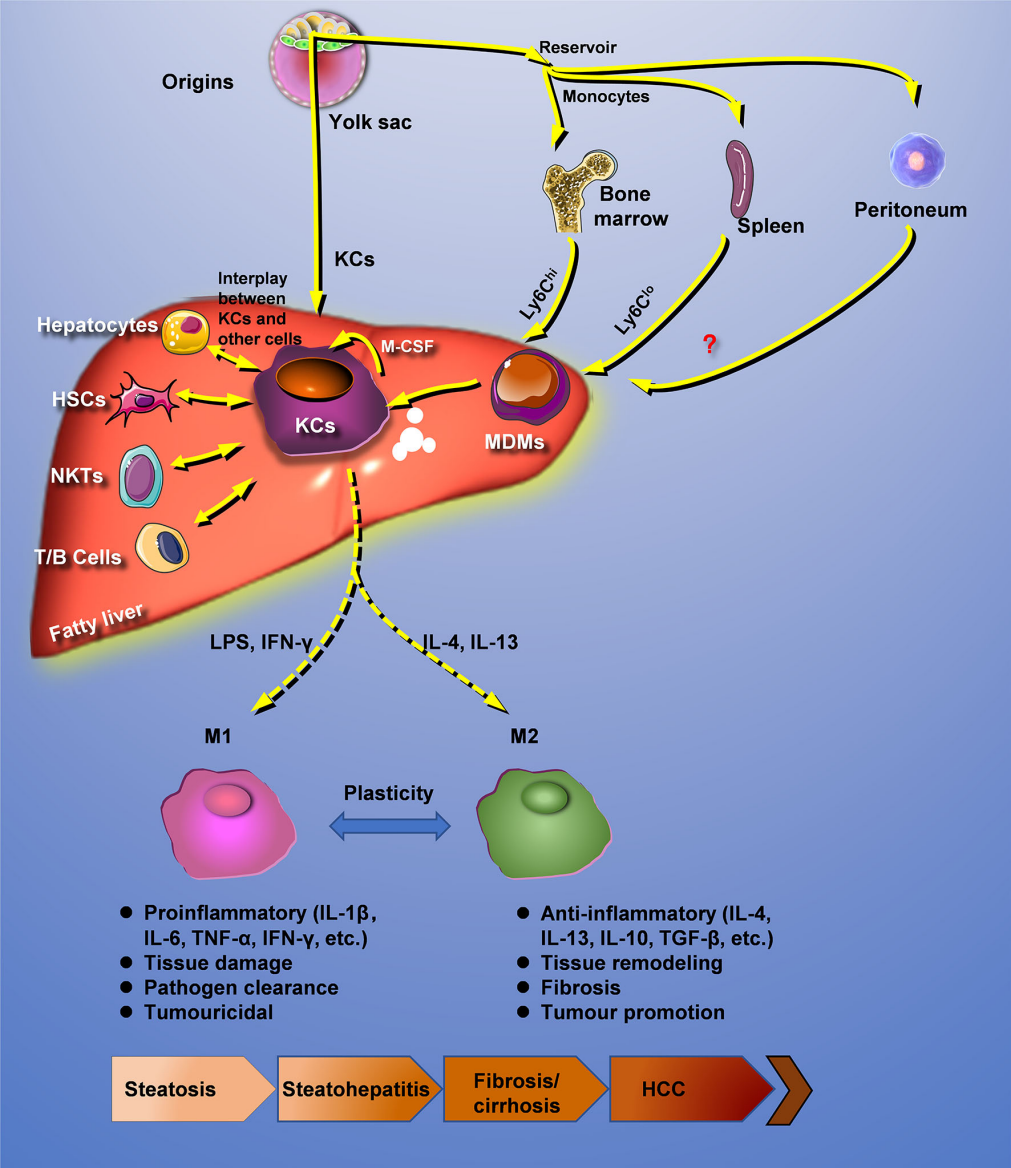
Crosstalk with other cells and reprogramming of hepatic macrophages. Under pathologic conditions, hepatic macrophages are interacted with other cells and reprogrammed. HCC, hepatocellular carcinoma; HSCs, hepatic stellate cells; KCs, Kupffer cells; MDMs, mononuclear-derived macrophages.

Hepatic macrophages are interacted with other cells and reprogrammed under pathologic conditions. In fatty liver disease, distinctly heterogeneous populations of macrophages can recognize extracellular stimuli through PRRs, including membrane-bound Toll-like receptors (TLRs) and cytoplasmic nucleotide-binding oligomerization domain-like receptors (NLRs), resulting in the secretion of a large amount of inflammatory cytokines, chemokines, and other reactive molecules such as ROS and NO (50). In addition, macrophages could also participate in the progression of fatty liver disease through lipotoxicity, glucotoxicity, and iron poisoning (**Figure 1**).

### Crosstalk Between Hepatic Macrophages and Surrounding Cells in Fatty Liver Disease

KCs are located in the center of hepatic sinusoids; therefore, it is possible that KCs are in intimate contact with the surrounding immune and parenchymal cells in the liver (51). KCs affect lipid metabolism in hepatic parenchyma cells through cell–cell crosstalk. In patients with fatty liver at an early stage, an increase in portal macrophages in liver biopsy sections was the earliest change detected (52). In an HFD-induced murine fatty liver disease, IL-1β released from KCs promotes hepatic steatosis by inhibiting PPARα activity in hepatocytes (47). The released TNF-α activates Caspase-8 in hepatocytes and triggers hepatocyte apoptosis by binding to TNF receptor 1 (53). The increased circulating IL-6 contributes to the development of obesity-associated IR in hepatocytes (54). Additionally, KCs can engulf apoptotic hepatocytes *via* efferocytic clearance triggered by phosphatidylserine (PS) signals. Recently, our group reported that the PS receptors T cell immunoglobulin mucin domain-containing molecule 3 (Tim-3) and Tim-4 are elevated in liver macrophages in murine steatohepatitis, and their knockout leads to an increased production of ROS, IL-1β, and IL-18 in macrophages, with aggravation of steatosis and inflammation. However, the underlying mechanisms of macrophage-mediated efferocytosis in fatty liver disease require further exploration (55, 56).

KCs can also regulate hepatic stellate cell (HSC) activation by cytokines and chemokines. A co-culture system of macrophages and hepatocytes suggests that macrophages activate the NF-κB signaling pathway in HSCs *via* the increased secretion of IL-1β, TNF-α, and IL-6 and the upregulated expression of the tissue inhibitor of metalloproteinase 1 in HSCs, which promotes the progression of hepatic fibrosis (57). Activated KCs display a strong acceleration of CC chemokine-induced HSC migration and recruitment through CCL2 and CCL5 production (58). KCs also activate HSCs through TGF-β and platelet-derived growth factor (PDGF), which increases the expression of collagen and accelerates fibrosis progression by trans-differentiating HSCs into myofibroblast phenotype (59–61).

In murine steatohepatitis models, KC secretion of the chemokines CCL2, CXCL10, and TNF-related apoptosis-inducing ligand (TRAIL) is upregulated to recruit monocytes and to trigger monocyte differentiation into KCs. This process is characterized by the over-expression of MMP-12, MMP-13, and insulin growth factor 1 to ameliorate liver injury and fibrosis by degrading the matrix (62, 63). The ability of KCs from HFD-induced mice to recruit CD4^+^ T lymphocytes and B cells is increased (64). Hepatic macrophages promote neutrophil adhesion to liver sinusoidal endothelial cells (LSECs) *via* TLR4 (65) and increase neutrophil recruitment *via* chemokines, such as CXCL1, CXCL2, and CXCL8 (66). KCs can also affect the number and activation of natural killer T (NKT) cells in various ways. KC-derived chemokine CXCL16 recruits CXCR6-expressing NKT cells to accentuate inflammation and fibrosis in the liver (46). The interaction between Tim-3^+^ KCs and Gal-9 promotes NKT cell proliferation by IL-15 secretion in an HFD-induced murine fatty liver disease (67). In addition, IL-12 released by KCs is associated with the reduction of hepatic NKT cells in the liver of choline-deficient diet-induced mice (68). In addition, PA exposure-activated macrophages present exogenous antigens to NKT cells through CD1d, resulting in excessive activation and apoptosis of NKT cells in HFD-fed mice (69).

It is evidenced that KCs can regulate the function of other cells in the liver. However, they are also influenced by hepatocytes and surrounding cells in the microenvironment of fatty liver disease. Hepatic macrophages internalize extracellular vesicles (EVs). Lipotoxic hepatocyte-derived EVs are enriched with CXCL10, which induces the hepatic recruitment of monocytes, depending on JNK and mixed lineage kinase 3 pathway, in addition to ceramide-containing EVs (70, 71). Moreover, lipotoxic hepatocytes can release active integrin β1-containing EVs to mediate monocyte adhesion to LSECs and inflammation in murine steatohepatitis (72). The injured hepatocytes can also release HMGB1-containing EVs, which mediate mitochondrial damage through the TLR4-JNK pathway and induce inflammation by activating KCs (73). In addition, CCL2 released from lipotoxic hepatocytes recruits monocytes to the liver by binding to CCR2 on monocytes in the process of liver injury (69, 70). The leaked cholesterol crystals from apoptotic hepatocytes can be engulfed by KCs and activate the NLRP3 inflammasome in KCs, causing the production of pro-inflammatory cytokines (74). KC-mediated clearance of apoptotic bodies formed by hepatocytes promotes the production of death receptors, including Fas ligand and TNF-α, which further induces hepatocyte apoptosis, depending on a positive feedback loop (75). Damaged hepatocyte-derived mtDNA could be sensed by the stimulator of IFN genes (STING) in KCs to increase TNF-α and IL-6 production in MCD and HFD-induced murine steatohepatitis models (76). ATP released from damaged hepatocytes promotes NLRP3 inflammasome activation and IL-1β and IL-18 release by the P2X7 receptor on KCs (77). LSECs facilitate the hepatic recruitment of monocytes through the increased production of CCL2, and they could also display anti-inflammatory properties to prevent KC activation in the progression of fatty liver disease (78). In low-density lipoprotein receptor-deficient mice, increased myeloperoxidase secreted by neutrophils causes toxicity to macrophages and aggravates inflammation and insulin resistance (79). Consistent with this, myeloperoxidase deficiency reduces liver inflammation and improves IR in murine fatty liver disease (23). In addition, ROS and growth factors released from neutrophils enhance the M1-like macrophage function in promoting fibrosis by activating HSCs (80). Moreover, single-cell RNA sequencing results showed that activated HSCs regulate the functions of macrophages *via* HSC-derived stellakines, such as CCL11, CCL2, and CXCL2, in the livers of murine steatohepatitis (81).

Taken together, these findings indicate that macrophages participate in fatty liver disease by regulating the liver parenchymal cells, HSCs, and recruitment of monocytes and NKT cells. Conversely, infiltrating neutrophils and damaged hepatocytes also activate macrophages by secreting factors, which further aggravates the progression of fatty liver disease.

### Metabolic Reprogramming of Hepatic Macrophages in Fatty Liver Disease

In fatty liver disease, macrophages require metabolic reprogramming to meet the demands for energy and biosynthesis during the process of activation, while changes in metabolic patterns could switch the phenotype of macrophages. A glucose metabolic shift occurs during macrophage polarization. When exposed to LPS and IFN-γ, macrophages are polarized into the M1 phenotype, accompanied by enhanced glycolysis, increased lactic acid production, and activation of the pentose phosphate pathway (PPP). When stimulated by IL-4, IL-13, IL-10, or glucocorticoids, macrophages differentiate into the M2 phenotype and secrete the anti-inflammatory factor IL-10, which results in augmented oxidative phosphorylation (82, 83). A lipid metabolic shift also occurs during macrophage polarization. *In vitro* studies have shown that IL-4 treatment increases the fatty acid intake and fatty acid oxidation of macrophages; however, IFN-γ and LPS stimulation decreases fatty acid uptake and fatty acid oxidation (84).

In fatty liver disease, the regulation of lipid metabolism determines the macrophage phenotype. Saturated fatty acids promote macrophage differentiation toward the M1 phenotype by activating the NF-κB pathway and increase lipid synthesis by activating sterol regulatory element binding protein-1c (SREBP-1c) in fatty liver disease (85, 86). Fatty acids can regulate lipid metabolism by activating the nuclear transcription factor PPARs. Myeloid-specific PPARδ knockout mice display increased IR and the occurrence of hepatitis by inhibiting macrophage transition to the M2 phenotype (87). Recent evidence shows that hepatic retinoic acid receptor-related orphan receptor-α (ROR-α) promotes macrophage differentiation to the M2 phenotype through kruppel-like factor 4. Moreover, ROR-α-specific knockout macrophages aggravate lipid deposition in HFD-fed mice (88). Increasing evidence indicate that metabolic reprogramming can ameliorate steatosis by switching macrophages to the M2 phenotype (**Figure 1**).

### Hepatic Macrophages Participate in Fatty Liver Disease Progression Through TLRs

TLRs mainly recognize bacterial products derived from components of intestinal bacteria, such as LPS and peptidoglycan. The microenvironment of fatty liver disease upregulates TLR4 expression, increases intestinal permeability, and leads to a significant increase in serum LPS. These events lead to the activation of the MyD88-NF-κB signaling pathway and promote inflammatory cytokine secretion in both humans and mice with fatty liver disease (89–91). Moreover, KCs isolated from HFD-fed mice are more sensitive to LPS-induced activation (92).

High-mobility group protein B1 (HMGB1) can trigger TLR4 signaling pathway by promoting p38 phosphorylation, NF-κB translocation, TNF-α release, and polarization of M1-like macrophages (93, 94). The abundance of free fatty acids (FFAs) in the microenvironment of fatty liver disease activates inflammatory signaling pathways in KCs in a TLR2- and TLR4-dependent manner (95–97). Moreover, FFAs can also activate KCs by binding to TLR4 indirectly *via* fetuin-A (98, 99). The knockout of *MyD88* attenuates steatosis and hepatitis induced by a choline-deficient amino acid-defined (CDAA) diet in mice (100). HFD-induced fatty liver disease in mice with macrophage-specific *IKK-β* deficiency displays over-activation of the NF-κB pathway, insulin resistance, and hepatitis (101). IKK2 inhibition of the NF-κB pathway alleviates steatosis and inflammatory responses in murine steatohepatitis (102).

Unlike the surface receptors TLR2 and TLR4, TLR9 is confined primarily to the endosomes of macrophages. In HFD-fed mice, an increased level of mtDNA released from damaged hepatocytes is responsible for the activation of macrophage populations *via* TLR9 activation (103). The unmethylated CpG motif-containing bacterial DNA could also bind to TLR9 in KCs and promote IL-1β secretion. In CDAA diet-induced murine steatohepatitis, TLR9 knockout relieves hepatic steatosis, inflammation, and fibrosis by suppressing IL-1β secreted by KCs rather than hepatocytes and hepatic stellate cells (100). TLR9 deficiency also suppresses lipid deposition in HFD-fed mice (103).

**Figure 2:**
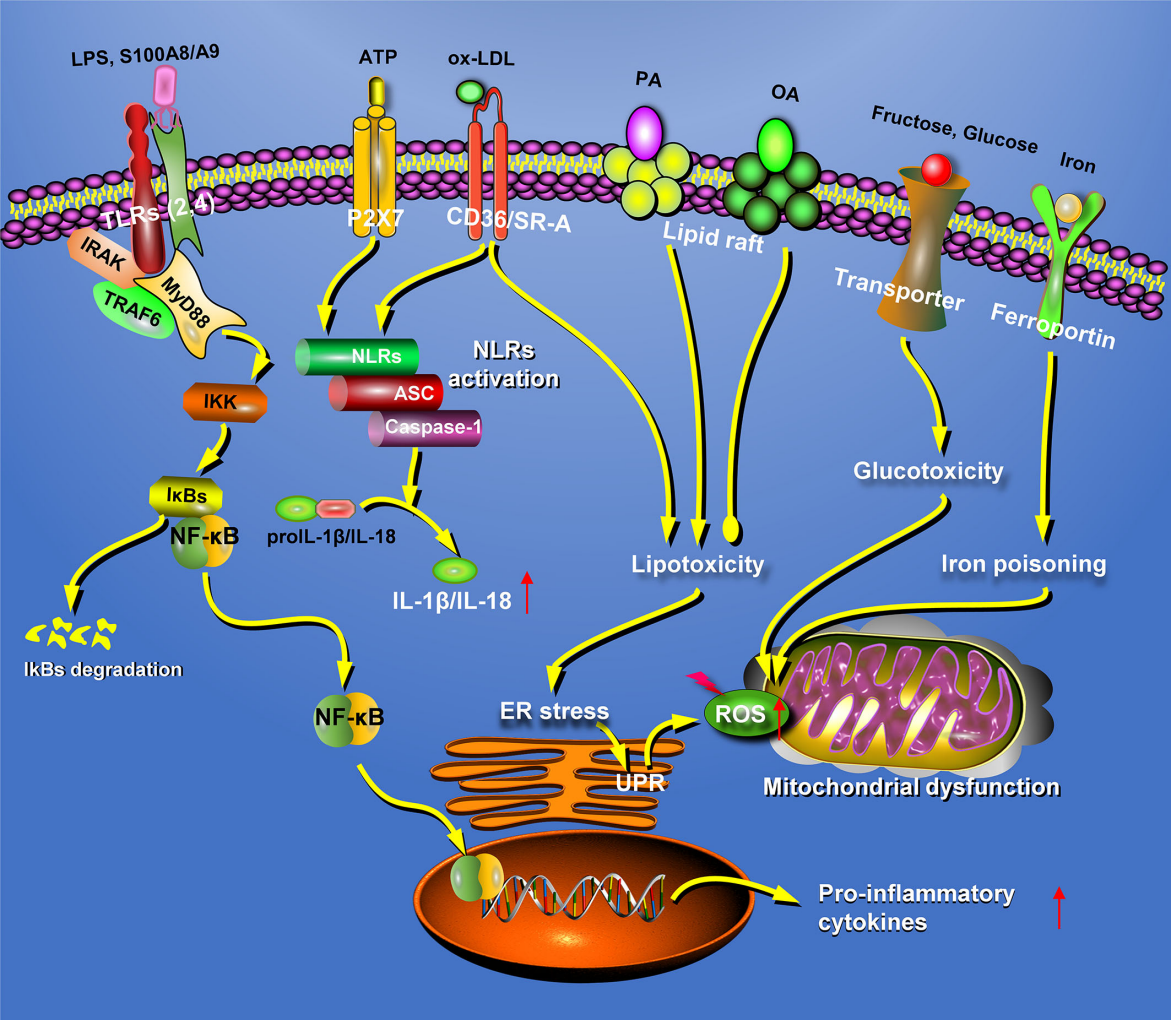
Hepatic macrophages participate in the pathogenesis of fatty liver disease in many different patterns. In fatty liver disease, the macrophages can recognize extracellular stimuli through pattern recognition receptors, including TLRs and NLRs, resulting in the secretion of inflammatory factors. In addition, macrophages could also participate in the progression of NAFLD through lipotoxicity, glucotoxicity, and iron poisoning. ATP, adenosine triphosphate; ER, endoplasmic reticulum; NLRs, nucleotide-binding oligomerization domain-like receptors; OA, oleic acid; PA, palmitic acid; P2X7, P2X purinoceptor 7; ROS, reactive oxygen species; S100A8, S100 calcium-binding proteins A8; SR-A, scavenger receptor-A; TLRs, Toll-like receptors; UPR, unfolded protein response.

Taken together, these results suggest that lipids activate macrophages through TLR pathways, thus promoting the development of fatty liver disease (**Figure 2**). Therefore, intervention of the TLR pathway might be an ideal strategy for the treatment of fatty liver disease in the future.

### Hepatic Macrophages Participate in Fatty Liver Disease Progression Through NLRs, Especially NLRP3 Inflammasome

NLRs are components of inflammasomes in the cytoplasm, and the responses to danger signals lead to inflammasome activation and IL-1β and IL-18 secretion. The NLRP3 inflammasome is currently the most extensively studied and best-characterized inflammasome in macrophages. Moreover, KCs are considered to be the main cell type responsible for NLRP3 inflammasome activation in the liver. It has been reported that Caspase-1 activity and mature IL-1β levels are significantly increased in steatohepatitis models (104), and NLRP3 inflammasome activation aggravates hepatic steatosis, liver inflammation, and fibrogenesis, supporting the contribution of NLRP3 inflammasome to pathogenesis of fatty liver disease (105). Accordingly, the inhibition of NLRP3 inflammasome by related gene knockout or specific inhibitors has been proposed as an effective therapeutic option for fatty liver disease (106, 107).

Classical NLRP3 inflammasome activation requires active Caspase-1. In this process, adenosine triphosphate (ATP) and P2X purinoceptor 7 (P2X7) receptors on KCs mediate the assembly of the NLRP3 inflammasome. Once activated, pro-Caspase-1 permits auto-cleavage and forms an active Caspase-1 p10/p20 tetramer, which cleaves pro-IL-1β and pro-IL-18 to generate mature IL-1β and IL-18. It has been reported that non-classical NLRP3 inflammasome activation also exists in KCs, requiring active Caspase-11. In this process, LPS from the cytoplasm binds directly to pro-Caspase-11 (108), or LPS binds to TLR4 in the endosome, which promotes pro-Caspase-11 synthesis through the TRAF3-IRF3-IFN pathway (109), resulting in the production of active Caspase-11 and Gsdmd. Active Gsdmd induces KC pyroptosis and promotes the secretion of IL-1β and IL-18 (108, 110–112). Caspase-1 and -11 depletion in macrophages attenuate liver inflammation by reducing the formation of cholesterol crystals and increasing cholesterol effusion (113). Notably, recent studies have shown that inflammasomes are released from virus-infected macrophages, which could provide a novel approach for preventing chronic inflammation (114).

In addition to TLRs and NLRs, hepatic macrophages are also regulated by other receptors. Recently, it was reported that bile acid (a regulator of glycolipid metabolism) inhibits inflammasome activation by binding to transmembrane G protein-coupled receptor 5 on KCs and upregulates the production of anti-inflammatory cytokines (115). In a high-fat- and high-cholesterol-diet-induced murine fatty liver disease, dietary advanced glycation end-product (AGE) activates the MyD88-NF-κB signaling pathway in KCs by upregulating the receptor for AGE (RAGE) (116). S100 calcium-binding protein A8 (S100A8) and S100A9 promotes inflammatory responses in macrophages in both RAGE- and TLR4-dependent manners (117, 118). Docosahexaenoic acid activates PPARγ and AMPKα by binding to GPCR120 on macrophages and decreases the expression of pro-inflammatory genes by suppressing the NF-κB pathway (119–121). Taken together, these data suggest that activation of the NLRP3 inflammasome can aggravate hepatic steatosis (**Figure 2**).

### Cytokines From Hepatic Macrophages in Fatty Liver Disease

Hepatic macrophages participate in the development of fatty liver disease by secreting multiple pro-inflammatory cytokines, including IL-1β, IL-6, and TNF-α. In mice with fatty liver disease, KCs are a major source of IL-1β. Enhanced IL-1β from activated M1-like macrophages could promote hepatic inflammation by upregulating ICAM-1 in LSECs to attract more neutrophils into the liver (122). IL-1β promotes steatosis by inhibiting fatty acid oxidation *via* PPARα and promotes TG synthesis, accumulation and lipid droplet formation in hepatocytes (43, 123). IL-1β can also promote hepatocyte apoptosis and aggravate liver fibrosis by activating NF-κB in HSCs (100). Increased IL-6 levels result in an enhanced risk of insulin resistance in patients with fatty liver disease (124). KCs secrete TGF-β and PDGF, which are potent mitogenic factors of HSCs and are vital for hepatic fibrosis progression (125). High levels of TNF-α contribute to inflammasome activation through the NF-κB pathway and aggravate inflammatory injury, insulin resistance, and steatosis (126, 127). Under FFA stimulation or an HFD-induced microenvironment of fatty liver disease, activated KCs release more TNF-α and IFN-γ (128). TNF-α increases hepatic cholesterol synthesis and suppresses its elimination, which results in a dramatic increase in LDL cholesterol and a decrease in HDL cholesterol (129, 130), while a TNF-α blocking antibody alleviates hepatic steatosis in ob/ob mice that have a leptin deficiency (131). In patients with fatty liver disease, TNF-α and IL-8 released from myeloid-derived immune cells, including KCs, DCs, and neutrophils, are positively correlated with the severity of fatty liver disease (132). However, clinical evidences show that treatment with a TNF-α-specific blocking antibody-CDP571 could not alleviate the symptoms of metabolic diseases (133), which may be related to the multiple sources of TNF-α from KCs (57), DCs (134), neutrophils (135), and broad effects in fatty liver disease. The above-mentioned data indicate that a large number of inflammatory cytokines secreted by activated KCs participate in the progression of fatty liver disease.

Activated KCs also participate in fatty liver disease by secreting a variety of chemokines to recruit mononuclear cells to the liver. Levels of CCL2 and CCL19 are increased in the serum of patients with fatty liver disease (136). In murine steatohepatitis, CCL2 binding to CCR2 on Ly6C^+^ monocytes (137) or CCL1 binding to CCR8 (10) results in more Ly6C^+^ monocyte recruitment into the liver, which further promotes the progression of hepatitis and liver fibrosis. Therefore, the infiltration of Ly6C^+^ monocytes has been identified as a key factor in the progression of steatohepatitis and hepatic fibrosis in mice (138, 139). CCR2^-/-^ or CCR2 inhibitor treatment in mice alleviates steatosis, inflammatory cell infiltration, and fibrosis (98, 99). It was also confirmed that the proportion of macrophages in the liver decreased by approximately 80% following the reinfusion of *CCR2^-/-^* monocytes (140). CCL2/CCR2 has been identified as chemokines that promote monocyte infiltration into the injured liver; therefore, CCL2/CCR2 is likely to be applied in clinics in the treatment of fatty liver disease in the future. In addition to CCL2, TRAIL, which is secreted by KCs, is also involved in the recruitment of monocytes (141). Monocytes are also recruited by CXCR3, CXCL10, or ceramide through sphingosine 1-phosphate in murine steatohepatitis (142–144).

These results indicate that activated KCs secrete many pro-inflammatory factors that aggravate the development of fatty liver disease (**Figure 2**).

### Lipotoxicity in Hepatic Macrophages Under Fatty Liver Disease

Under physiological conditions, lipids are responsible for maintaining intracellular metabolism, cell communication, inflammation regulation, and the membrane structural integrity in fatty liver disease. When the rate of fatty acid uptake and synthesis exceeds the rate of fatty acid removal, fatty acids induce cellular stress and lipid toxicity. The term “lipid toxicity” was proposed by Unger for the first time in 1994 when he described cell damage in the muscle of patients with type 2 diabetes and metabolic syndrome induced by toxic lipid molecules (145).

Increased TG levels and the upregulated expression of fatty acid synthesis-related genes, including carbohydrate response element binding protein 1 (*CHREBP1*), *PPARγ*, fatty acid synthase, fatty acid binding protein 2, fatty acid transporter 5, and DAG acyltransferase, lead to an excessive accumulation of lipids in KCs (64). Although TG accounts for the largest proportion of these over-synthesized lipids, TG is almost a non-lipotoxic molecule (146, 147). Although lipidomic analysis revealed that the total accumulation of non-toxic TG in the liver of HFD mice was significantly higher than that observed in ND mice, there was no significant difference in the deposition of TG in KCs. In murine steatohepatitis, TG synthesis inhibition ameliorates hepatic steatosis but aggravates liver injury and fibrosis (146).

A relatively small proportion of lipids, including saturated fatty acids, free cholesterol, DAG, ceramide, lysophosphatidylcholine, and bile acid, can be lipotoxic to KCs and hepatocytes (148–152). A sustained toxic lipid accumulation in KCs disrupts the structure of lipid rafts in the plasma and mitochondrial membrane and results in oxidative and endoplasmic reticulum (ER) stress. Under toxic lipid exposure, macrophages are polarized towards the M1 phenotype, with high levels of inflammatory cytokines (such as TNF-α, IL-6, and IL-1β) and secretion of chemokines (such as CCL2, CCL5, and CXCL10) (145). The most abundant fatty acids found in food and fatty liver are saturated fatty acids (PAs) and monounsaturated fatty acids (OA). The excessive accumulation of PA and FFAs in KCs activates the inflammatory signaling pathway (95), induces ER stress and mitochondrial injury, and increases ROS levels (153). However, OA and polyunsaturated fatty acids, such as omega-3 and omega-6, attenuate inflammation and lipotoxicity (154, 155). Saturated fatty acid-induced lipotoxicity in KCs exacerbates the development of fatty liver disease. Short-chain fatty acids enhance fatty acid oxidation and inhibit steatosis progression through PPARγ (156).

Normally, FFAs can be oxidized in the mitochondria, peroxisomes, and microsomes to produce ROS. In fatty liver disease, steatosis increases the efflux of FFAs into the liver, leading to elevated fatty acid β-oxidation and ROS production in the mitochondria. However, excessive ROS leads to mitochondrial dysfunction by reacting with polyunsaturated fatty acids in the mitochondrial membrane, which results in mitochondrial membrane injury, superoxide dismutase inactivation, mitochondrial DNA mutations, and fragmentation (157, 158). In addition, accumulation of misfolded proteins in the ER causes dysfunction and ER stress, which triggers the activation of the unfolded protein response (UPR). In patients with fatty liver disease, increased ER stress activates UPR through transducers inositol-requiring enzyme 1, protein kinase R-like kinase, and activating transcription factor 6, which promotes the expression of p53, release of cytochrome C from the mitochondria, and the activation of NF-κB, JNK, and CEBP signaling pathways in KCs, resulting in IR and apoptosis (159, 160).

In HFD-fed mice, the accumulation of toxic lipids in KCs, such as free cholesterol, cholesterol ester, DAG, and ceramide, is much higher than that in ND mice. Accumulating evidence indicate that lipotoxicity caused by excessive cholesterol accumulation in KCs leads to foam-like cell formation and accelerated fatty liver disease development from simple steatosis to steatohepatitis. Cholesterol uptake by KCs occurs in two ways: LDL receptor (LDLR)-mediated endocytosis and modified LDL uptake by scavenger receptors (SRs). The native LDLs binding to LDLR on KCs promote the lysosomal degradation of LDL into free cholesterol. Increased levels of free cholesterol reduce the intake of cholesterol by inhibiting LDLR and producing lipid-loaded foam-like KCs containing cholesterol crystals. Modified LDLs, such as ox-LDL, are ingested by SR-A and CD36 on KCs, resulting in excessive cholesterol accumulation in lysosomes, NLRP3 inflammasome activation, and foam-like KCs in NASH (161). Moreover, intracellular cholesterol does not regulate SR-A expression, which further increases the number of foam-like KCs and accelerates the development of fatty liver disease. Foam-like KCs secrete chemokines to recruit monocytes and neutrophils and TNF-α and TGF-β to activate hepatic stellate cells, which transform them into myofibroblasts, resulting in hepatic fibrosis (162, 163).

The storage of cholesterol in the mitochondria is increased in human steatohepatitis as evidenced by upregulated mitochondrial cholesterol transporters and steroidogenic acute regulatory protein, but a small amount of cholesterol is found in the cell membrane and ER (164). In rats fed a choline-deficient high-cholesterol diet, mitochondrial function is impaired by the accumulation of free cholesterol and increased sensitivity to TNF-α, leading to Fas-mediated liver injury (165). Cholesterol-lowering agents, such as 2-hydroxypropyl-β-cyclodextrin, could further promote cholesterol efflux from lysosomes to alleviate liver inflammation in murine steatohepatitis (166). In *Ldlr^−/−^* mice, *SRA^-/-^/Cd36^-/-^* bone marrow transplantation partially alleviated high-fat- and high-cholesterol-diet-induced inflammation and fibrosis (167). Steatohepatitis induced by an HFD diet combined with ox-LDL also illustrates the important role of ox-LDL in the progression of fatty liver disease. Taken together, the long-term accumulation of toxic lipids in macrophages accelerates the progression of fatty liver disease (**Figure 2**).

### Glucotoxicity in Hepatic Macrophages Under Fatty Liver Disease

Fatty liver disease progression is associated not only with lipids but also with sugar and glucose transporters (GLUTs), which affect the activity or phenotype switching of macrophages. Glucose and fructose are the two most dominant monosaccharides. However, only fructose is metabolized in the liver (168) and displays a stronger lipogenesis effect (10%) than glucose (2%) (169). Compared with healthy individuals, patients with fatty liver disease have a higher fructose intake (170). A high-sugar diet could further fuel HFD-induced fatty liver disease progression. Evidence reveals that fructose can promote the progression of fatty liver disease by regulating lipase activity, increasing intestinal permeability and motility *via* TLR4 on KCs (171) and enhancing the interaction with thioredoxin-interacting protein in macrophages. Enhanced nuclear transcription factor SREBP-1c and CHREBP1 promote the *de novo* synthesis of lipids in the liver (172). Thioredoxin is shuttled into the mitochondria to mediate NLRP3 inflammasome activation and IL-1β, IL-18, and ROS production (173).

In fatty liver disease, increased glucose transporter GLUT1 promotes M1-like macrophage polarization by upregulating the PPP (174). In addition, hypoxia increases glucose uptake by GLUT3 in macrophages, which increases the *de novo* synthesis and deposition of lipids and promotes the progression of fatty liver disease (175). In summary, high glucose levels polarize macrophages to the pro-inflammatory M1 phenotype and promote the progression of fatty liver disease (**Figure 2**).

### Iron Poisoning of Hepatic Macrophages in Fatty Liver Disease

Hepatic iron overload contributes to hepatic inflammation by increasing hepatic cytokine expression in a HFD plus high-iron-induced rat model (176), while hepatic iron depletion by deferoxamine treatment in ob/ob mice improved hepatic steatosis by upregulating lipid metabolism-related genes as well as reducing free radical formation and pro-inflammatory cytokines (177). Although iron overload leads to macrophage polarization toward the M1 phenotype and a significant decrease in prominent regulators of M2 activation, such as PGC-1β, PPARγ, and KLF4, and reduced phosphorylation of STAT6 (178), iron-overloaded hepatic macrophages activate a novel signaling pathway partially consisting of MEK1-TAK1-PI3K-IκB kinase (179).

In a study of 849 patients with steatohepatitis, more than 34% of patients displayed high amounts of iron deposition in liver biopsies (180), and approximately 34% of patients with fatty liver disease showed a dysfunction in the metabolism of iron overload (181). In addition, serum transferrin levels are increased in patients with fatty liver disease (182). Abnormal phagocytosis of erythrocytes by hepatic KCs possibly leads to the accumulation of hemoglobin iron in the liver and triggers oxidative stress (183).

Collectively, these findings suggest that iron poisoning of hepatic macrophages is involved in fatty liver disease (**Figure 2**), but the mechanisms of iron accumulation in KCs remain unclear and require further investigation.

## Discussion

Several studies in both human and animal models have shown that hepatic macrophages play a central role in the development and progression of fatty liver disease. In the microenvironment of fatty liver disease, any signal of DAMPs, PAMPs, lipotoxicity, or glucotoxicity could trigger KC activation or polarization through the TLR or NLR signaling pathways, resulting in the increased secretion of inflammatory cytokines and chemokines and imbalanced metabolic reprogramming. The released cytokines facilitate the communication between KCs and other cells, including parenchymal cells, HSCs, NK cells, and NKT cells, and the activation of these cells in the liver, while secreted chemokines foster more monocyte infiltration into the liver, where they can differentiate into KCs in a positive loop manner. In addition, metabolic reprogramming leads to disorders in glycolysis, lipid synthesis, and iron metabolism. All these abnormalities collectively contribute to steatosis, inflammation, and fibrogenesis in the development of fatty liver disease. The current strategies for targeting macrophages to treat fatty liver disease mainly include the inhibition of macrophage activation (*e*.*g*., *via* inhibiting the inflammasome assembly), regulation of macrophage polarization (*e*.*g*., *via* promoting polarization into the M2 phenotype through nanoparticles), inhibition of monocyte recruitment and infiltration (*e*.*g*., *via* suppressing the expression of chemokines like CCL2, CCL10, or CCL3), and amelioration of toxic lipid accumulation (*e*.*g*., *via* promoting lipolysis, FFA efflux, and transformation to nontoxic TG) (184–186). Various medications currently targeting macrophage for fatty liver disease are under clinical evaluation in humans. These medications include cenicriviroc, selonsertib, emricasan, GR-MD-02, IMM-124E, JKB-121, SGM-1019, tropifexor, GS-0976, GS-9674, and lanifibranor (9, 10). Further in-depth investigation of hepatic macrophages will help develop novel strategies for the treatment of fatty liver disease and related chronic liver diseases in the future.

## Author Contributions

LX and WL wrote and revised the manuscript. FB, YX, XL, and CM collected the related papers and helped to draft and revise the manuscript. LG participated in the design of the manuscript and was the major contributor. All authors contributed to the article and approved the submitted version.

## Funding

This work was supported by the National Natural Science Foundation of China (nos. 81971480, 81670520, and 81902921), the joint fund project of the Natural Science Foundation of Shandong Province (ZR2019LZL013), the Taishan Scholarship (no. tspd20181201), and the Shandong Provincial Key Innovation project (no. 2018YFJH0503). The authors would like to express their thanks for the support from the Collaborative Innovation Center of Technology and Equipment for Biological Diagnosis and Therapy in the Universities of Shandong.

## Conflict of Interest

The authors declare that the research was conducted in the absence of any commercial or financial relationships that could be construed as a potential conflict of interest.

## Publisher’s Note

All claims expressed in this article are solely those of the authors and do not necessarily represent those of their affiliated organizations, or those of the publisher, the editors and the reviewers. Any product that may be evaluated in this article, or claim that may be made by its manufacturer, is not guaranteed or endorsed by the publisher.
